# Inhibiting the NLRP3 Inflammasome With Methylene Blue as Treatment Adjunct in Myelodysplasia

**DOI:** 10.3389/fonc.2018.00280

**Published:** 2018-07-27

**Authors:** Richard E. Kast

**Affiliations:** IIAIGC Study Center, Burlington, VT, United States

**Keywords:** bone marrow, inflammasome, innate immune response, methylene blue, myelodysplasia, pyroptosis

## Abstract

Myelodysplasia refers to a group of clonal hematopoietic neoplasms characterized by genetic heterogeneity, different clinical behaviors and prognoses. Some of this group of bone marrow failure conditions have known external causes, some are of unknown origin. Within marrow, intracellular, and extracellular elements of the innate immune system are activated and contribute to creation of multiple cytogenetic abnormalities and are central to the mode of hematopoietic cell failure. Basiorka et al. showed that NLRP3 inflammasome activity is essential to the innate immune system's destruction of marrow hematopoietic cells commonly in myelodysplasia. In April 2018 Hao et al. reported that methylene blue inhibits rat NLRP3 inflammasome function. Methylene blue has been in continuous use in humans for over a century. It is associated with an eminently benign side effect profile in human use. If as in rodents, methylene blue also inhibits NLRP3 inflammasome function in human myelodysplasia a trial of adjunctive methylene blue treatment in transfusion dependent, low risk myelodysplasia where marrow inflammation and apoptosis predominates, would be worth trying.

**HIGHLIGHTS**

- Cytogenetic abnormalities and innate immune activation are seen in myelodysplasia
- The NLRP3 inflammasome is a core element generating marrow failure of myelodysplasia
- In April 2018 methylene blue was reported to potently inhibit NLRP3 inflammasome function
- Methylene blue has benign side effects and has been in human use for a century
- Study of methylene blue treatment of myelodysplasia would be a low-risk intervention

## Introduction—myelodysplasia

Myelodysplasia (MDS) refers to a group of clonal hematopoietic neoplasms characterized by genetic heterogeneity, differences in clinical behavior and prognosis, the incidence of which rises sharply with age (1). The clinical picture is variable, encompassing hematopoietic failure syndromes with pancytopenia, hypocellular dysplastic marrow, ineffective hematopoiesis, marrow stem cell macrocytosis, production of aberrant clones, a high marrow cell apoptosis rate (particularly in early stage disease), and multiple gross cytogenetic abnormalities (2, 3). A large number of different mutations are seen even within histologically homogeneous MDS patients, particularly in genes. related to RNA splicing machinery (4). Immunophenotyping with flow cytometry can provide indicators of low risk MDS (5) and is an important aspect to MDS subclassification (6).

Clinical heterogeneity need not be reflected by mutational heterogeneity (7). Seventeen percentage of people presenting with pancytopenia will be found to have MDS on marrow exam (8). Although MDS can run a smoldering course, an accelerated stage can supervene at some point. Differentiating therapies for (a) low risk MDS with repeated erythropoietin, lenalidomide, or various drugs to diminish immune mediated or inflammation related hematopoietic cell apoptosis, and (b) drugs for high risk MDS like azacitidine, decitabine, dose cytarabine, or marrow cytotoxic chemotherapy (7, 9, 10) leave room for improvement since across all MDS subtypes overall mortality at 5 years remains around 50% (1).

Bone marrow transplantation can be curative but carries its own morbidity and mortality and many MDS patients aren't eligible for transplantation (7). This paper presents the rationale for a currently available low-risk adjunct compatible with current treatment options to hopefully improve MDS prognosis.

Association of inflammation and chronic immune stimulation has long been associated with MDS, with destructive inflammation feedback amplification loops active in disease progression. Difficulties in modeling bone marrow niche in which MDS develops are well-known, limiting drug screening applicable to MDS. Below I show how a recent discovery about an old medicine might be able to clinically inhibit a key link in MDS pathophysiology, thereby retarding MDS progression.

## Inflammasome

As part of innate immunity, a wide array (dozens) of intracellular sensors are built-in to mammalian cells detect intracellular pathogens or bacterial products (11). The 10 human Toll-like receptors (TLR) tend to be on cell outer surface. Retinoid acid-inducible gene-I (RIG-I)-like receptors, and nucleotide oligomerization domain (NOD) -like receptors (NLR) are classes of such detectors that tend to be intracellular. NLR form an essential element of epithelial integrity protection, particularly along the gut.

Pathogen-associated molecular patterns (PAMPS) are commonly encountered lipids, sugars, or peptides found in pathogens that would not otherwise be present. Mammals have pre-existing receptors that when stimulated by their cognate molecular pattern—a PAMP—set in motion various defensive physiology changes. Receptors for PAMPS constitute important elements of the non-adaptive, innate immune system.

PAMPS' presence triggers NLRs to associate in multiprotein complexes called inflammasomes that mediate caspase-1 activation and cytokine secretion (12, 13). Caspase-1 converts pro-IL-1 beta to active 17.5 kDa. IL-1 beta and can lead to inflammation generation or to a specialized form of cell death or sequentially to both.

Nucleotide Binding Domain Leucine-Rich Repeat-Containing Receptor, Pyrin Domain-Containing-3 (NLRP3) Inflammasome is one such innate receptor complex (13).

NLRP3 itself acts both as an innate immune system sensor for a variety of intracellular PAMPS as well as an upstream link in triggering an inflammation response (13), the multiprotein macromolecular inflammasome forming in response to intracytoplasmic PAMPS' recognition.

But caspase-1 activation by NLRP3 inflammasome formation can, and in MDS does, lead to hematopoietic stem cell death (14–16). Caspase-1 is 200 times higher in MDS hematopoietic stem cells compared to normal (14).

The NLRP3 is one of dozens of NLRs. The NLRP3 macromolecular complex forms a critical convergence point in generating the swelling, DNA fragmentation, and caspase 1 activation characteristic of a subgroup of MDS marrows (15, 16). Cytosolic NLRP3 activation and the flood of cytokines consequent to its activation play a major role in death of in hematopoietic stem/progenitor cells seen in MDS.

Although the full pathophysiology of MDS remains today unexplained, Sallman et al showed that inhibition of the NLRP3 inflammasome could restore effective hematopoiesis in (16) in MDS. Below I show how this might be achieved today with an FDA, EMA, and Health Canada approved drug—methylene blue (MB, same as methylthioninium chloride USP, available today as Proveblue™ or from Star Pharmaceuticals (http://www.starpharm.com/).

## Methylene blue

Beginning with Paul Ehrlich in the late 1800's (17) continuing to today, as of 2018 (18–20), MB has been used to treat malaria. Today's clinical use of MB however is mainly (i) for treating methemoglobinemia (21), (ii) as a marker in finding compartment leaks and fistulae, (iii) in treating ifosfamide encephalopathy, and (iii) in reversal of perioperative vasoplegic after or during coronary artery bypass surgery (22, 23). Side effects from MB are few, transient, and mild (20, 24).

MB can enhance mitochondrial function. Under some circumstances MB circumvents mitochondrial complexes 2 and 3 by accepting an electron from complex 1, then delivering it to cytochrome C (20, 25).

Of central importance to MDS, three recent independent studies showed that MB inhibits NLRP3 inflammasome function. Ahn et al. showed that MB inhibited LPS stimulated increase in murine NLRP3 function (26). Lin et al. showed that MB inhibited murine cord transection initiated NLRP3 activation (27). Hao et al. showed that MB inhibited NLRP3 assembly and function in streptozocin murine model of diabetic retinopathy (28).

## Indirect evidence

We have only indirect evidence MB indeed inhibits NLRP3 activity *in vivo*. MB is clearly effective at stopping cerebral malaria in a murine model where NLRP3 is known to play a pivotal pathogenic role (29, 30). Of note this effect is independent of MB's anti-plasmodia effects (29, 30) with a parallel anti-inflammation action in minocycline's effect in cerebral malaria as predicted in 2008 and experimentally affirmed in a murine model in 2017 (31, 32).

Post-traumatic stress, when severe is associated with activated inflammation pathways and documented downstream cytokines known to be related to NLRP3 activation (33–35). As a consequence of these associations, a clinical trial of oral MB during standard exposure treatment of post-traumatic stress disorder showed benefit beyond that of exposure treatment plus placebo (35).

## Conclusion

This short note combines, (A) the 2015 and 2016 observations of Basiorka et al. (14, 15) and 2016 work of Sallman (16)—all three reporting that NLRP3 inflammasome constitutes a final common effector node driving a core element of the pathophysiology of MDS with, (B) the 2017 data sets of Ahn et al. (26) and of Lin et al. (27) plus the 2018 report of Hao et al. (28)—all three reporting that the NLRP3 inflammasome can be inhibited by the century old drug MB.

Adjunctive treatment of MDS might best be explored in early phase, low risk disease where apoptosis predominates the marrow pathology picture (36).

Thus a cheap, low risk adjuvant treatment option, MB, is potentially available now that might retard MDS progression.

## Author contributions

The author confirms being the sole contributor of this work and approved it for publication.

### Conflict of interest statement

The author declares that the research was conducted in the absence of any commercial or financial relationships that could be construed as a potential conflict of interest.

## Abbreviations

MDS: myelodysplasic syndrome
NLR: nucleotide oligomerization domain (NOD) -like receptors
NLRP3, Nucleotide Binding Domain Leucine-Rich Repeat-Containing Receptor: Pyrin Domain-Containing-3
PAMPS: Pathogen-associated molecular pattern
TLR: Toll-like receptors.
